# Changes in androstenedione, dehydroepiandrosterone, testosterone, estradiol, and estrone over the menopausal transition

**DOI:** 10.1186/s40695-017-0028-4

**Published:** 2017-10-17

**Authors:** Catherine Kim, Siobàn D. Harlow, Huiyong Zheng, Daniel S. McConnell, John F. Randolph

**Affiliations:** 1 Departments of Medicine and Obstetrics & Gynecology, University of Michigan, 2800 Plymouth Road, Building 16, Room 430W, Ann Arbor, MI 48109 USA; 2 Department of Epidemiology, University of Michigan, Ann Arbor, MI USA; 3 Department of Obstetrics & Gynecology, University of Michigan, Ann Arbor, MI USA

**Keywords:** Dehydroepiandrosterone-sulfate, Androstenedione, Testosterone, Estrone, Menopause

## Abstract

**Background:**

Previous reports have noted that dehydroepiandrosterone-sulfate (DHEAS) increases prior to the final menstrual period (FMP) and remains stable beyond the FMP. How DHEAS concentrations correspond with other sex hormones across the menopausal transition (MT) including androstenedione (A4), testosterone (T), estrone (E1), and estradiol (E2) is not known. Our objective was to examine how DHEAS, A4, T, E1, and E2 changed across the MT by White vs. African-American (AA) race/ethnicity.

**Methods:**

We conducted a longitudinal observational analysis of a subgroup of women from the Study of Women’s Health Across the Nation observed over 4 visits prior to and 4 visits after the FMP (*n* = 110 women over 9 years for 990 observations). The main outcome measures were DHEAS, A4, T, E1, and E2.

**Results:**

Compared to the decline in E2 concentrations, androgen concentrations declined minimally over the MT. T (β 9.180, p < 0.0001) and E1 (β 11.365, p < 0.0001) were higher in Whites than in AAs, while elevations in DHEAS (β 28.80, *p* = 0.061) and A4 (β 0.2556, *p* = 0.052) were borderline. Log-transformed E2 was similar between Whites and AAs (β 0.0764, *p* = 0.272). Body mass index (BMI) was not significantly associated with concentrations of androgens or E1 over time.

**Conclusion:**

This report suggests that the declines in E2 during the 4 years before and after the FMP are accompanied by minimal changes in DHEAS, A4, T, and E1. There are modest differences between Whites and AAs and minimal differences by BMI.

## Background

The menopausal transition (MT) represents a marked shift in women’s sex steroid profile, of which changes in estradiol (E2) are the best studied [1]. On average, women’s E2 concentrations begin to change more rapidly about 2 years prior to the final menstrual period (FMP) and stabilize several years after the FMP [2]. The rapidity of decline and average E2 levels may be predicted by race/ethnicity and body mass index (BMI) at the beginning of the transition [3, 4]. The most pronounced differences occur between African-American (AA) and White women, the former group having more gradual changes than the latter group [4]. Presumably in part due to adipose tissue production of E2, women with higher BMI have more gradual changes than women with lower BMI [3, 4].

The adrenal gland is the primary source of dehydroepiandrosterone-sulfate (DHEAS) and androstenedione (A4) and also contributes to circulating testosterone (T) [5]. Aromatase catalyzes A4 and T into estrogens, i.e. A4 into estrone (E1) and T into estradiol (E2). Previous reports have suggested that, prior to the FMP, adrenal DHEAS production increases even as peripheral E2 decreases [6–10]. As adrenal sex hormones exist in equilibrium with ovarian sex hormones in the peripheral circulation, it is plausible that adrenal hormone metabolism also changes over the MT [11]. This is consistent with the hypothesis that increasing adrenal sex hormone production and aromatization may be concurrent with decreasing ovarian estrogen production [12]. It is also possible that DHEAS production may also eventually decline over time resulting lower peripheral A4 and E1 concentrations.

Few longitudinal studies examine changes in a comprehensive array of adrenal sex hormones across the MT. Since concentrations of circulating DHEAS increase in the 5th decade of life [6–10] and concentrations among women in their 8th decade of life are low [13], DHEAS must decline in the postmenopause. However, it is uncertain when in the postmenopause this might occur. In addition, few reports examine concentrations of A4 or E1 during the MT and whether ratios of A4:E1 change over the MT, consistent with changes in aromatase activity or consistent with increased A4 production and concomitant increases in aromatization. No reports examine whether E1 concentrations change across the MT. In addition, studies have not examined whether these patterns differ by BMI, as has been reported for E2, or between Whites and AAs.

Therefore, using data from the Study of Women’s Health Across the Nation (SWAN), we characterized serum adrenal and ovarian sex steroid changes over the MT. We assessed concentrations of DHEAS, A4, T, E2, and E1 annually in the 4 years before and the 4 years after the FMP. We assessed whether concentrations changed in relation to the FMP during this time period and whether patterns differed between White and AA race/ethnicity, and BMI. We hypothesized that concentrations of DHEAS and A4 would increase slightly over the 4 years prior to and after the FMP, consistent with augmented adrenal androgen production. We hypothesized that AA women would be less likely to have adrenal sex hormone changes over the MT, as previous reports have suggested that AA women have less fluctuation in DHEAS concentrations than White women [4]. We also hypothesized that women with higher BMI would be more likely to have more gradual increases in DHEAS and A4 over the MT, since previous SWAN reports have suggested that women with higher BMI have more gradual declines in E2 than women with lower BMI [3].

## Methods

The study protocol of SWAN has been described previously: briefly, eligibility criteria for the SWAN cohort study enrollment included the following: age 42–52 years, no surgical removal of uterus and/or both ovaries; not currently using exogenous hormone medications that were known to affect ovarian function; at least one menstrual period as well as one of the following five other racial/ethnic groups. These groups included women who were White, AA, Chinese and Japanese, and Hispanic. A total of 3302 women were recruited. Institutional review boards approved the study protocol at each site; signed, written informed consent was obtained from all participants. The current study included a subsample of White and AA women who met inclusion criteria. We focused upon these 2 racial/ethnic groups as they had sufficient numbers of subjects with a documented final menstrual period (FMP) and complete hormone data for 4 years before and after the FMP, they were the largest number of participants in SWAN, the largest racial/ethnic differences in sex steroids have previously been observed between these 2 populations, and funds restricted examination of other racial/ethnic groups [3].

Other inclusion criteria included having a BMI of 22–30 kg/m^2^, a natural FMP i.e. no history of hysterectomy or oophorectomy, no exogenous hormone therapy use, and at least 9 sequential annual samples spanning 4 years before and 4 years after the FMP, for a total of 110 women with 990 observations. Compared to White participants in SWAN generally, White women in the current report were similarly aged at baseline, were more likely to report excellent or very good self-reported health, and had similar smoking status. Compared to AA participants who did not meet inclusion criteria, AA women in the current report had similar age, self-rated health, and smoking status. Due to the inclusion criteria designed to limit outliers of BMI, both White and AA women in the current report had lower BMI than women who did not meet inclusion criteria.

Annual fasting blood samples were collected. Two attempts were made to collect a follicular phase sample. When follicular phase samples were not available or when a woman stopped menstruating, a random fasting sample was collected within 90 days of the baseline recruitment date. All serum hormones were measured at the CLASS/RSP Central Laboratory at the University of Michigan (Ann Arbor, MI). A4 was measured using a commercially available enzyme-linked immunosorbent assay (ELISA) from Diagnostic Systems Laboratories (DSL). The assay measures analyte concentrations from 0.1 to 10 ng/mL with a minimum detectable concentration of 0.1 ng/mL, and a sensitivity of 0.03 ng/mL. The inter-assay coefficient of variation (CV) is 3.9% at 0.98 ng/mL and 3.0% at 6.1 ng/mL. The intra-assay CV is 2.1% at 0.98 ng/mL, 1.3% at 6.1 ng/mL. DHEAS was measured using an automated, ACS:180-based chemiluminescent assay developed in the CLASS laboratory and based upon the Bayer Diagnostics ACS:180. The detection level of this assay is approximately 1.9 μg/dL. The intra-assay CV is 8.02% (*n* = 261) and inter-assay CV is 11.34% (53.32 μg/dL, *n* = 37) and 9.74% (250.21 μg/dL, n = 37). The CLASS laboratory modified the ACS:180 total testosterone chemiluminescent assay to measure with greater precision samples in the low ranges found in women in the peri- and postmenopause. To accomplish this, sample volume was increased while evaluating the consequences of this change on volumes of subsequent reagents. The limit of detection of this assay is <5.15 ng/dL. The limit of quantification (lowest reported value) is set at the lowest standard, 5.15 ng/dL. The intra-assay CV is 11.78% (24.4 ng/dL, *n* = 30), 4.6% (191.2 ng/dL, n = 30) and 9.1% (414.2 ng/dL, n = 30). The inter-assay CVs are 11.34% (53.3 ng/dL, *n* = 37) and 9.7% (250.2 ng/dL, n = 37). E1 was measured using a commercially available ELISA from DSL. This method features a wide dynamic standard range of 0.05 to 90 ng/mL, and a minimum detectable concentration of 0.01 ng/mL. Inter-assay CVs were 12.7% at 1.2 ng/mL and 10.8% at 9.2 ng/mL, and intra-assay CVs were 6.7% at 1.2 ng/mL and 2.9% at 9.2 ng/mL. E2 concentrations were measured using the Estradiol-6 III immunoassay performed on the ADVIA Centaur instrument (Siemens HealthCare Diagnostics). Inter-assay CVs are 11.0% (102.9 pg/mL) and 7.0% (225.9 pg/mL) and 3.8% (615.9 pg/mL). Intra-assay CVs are 3.9% (102.9 pg/mL), 5.0% (225.9 pg/mL) and 1.4% (615.9 pg/mL). Follicle stimulating hormone (FSH) was measured with a two-site chemiluminescence (sandwich) immunoassay with a minimum detectable concentration of 0.3 mIU/mL. Inter- and intra-assay CVs are 8.1% and 3.5%, respectively.

### Statistical analyses

**Fig. 1:**
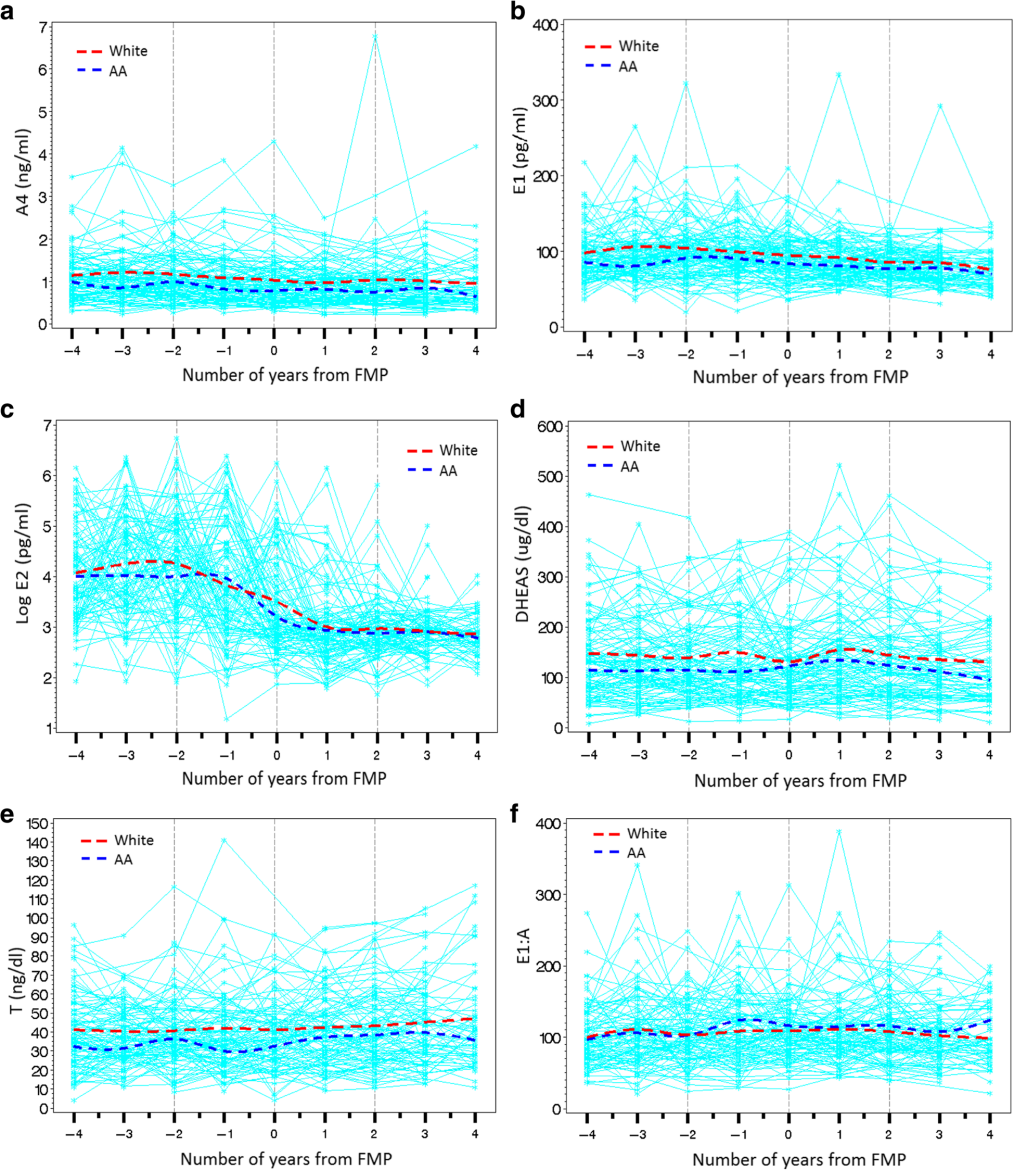
Concentrations of androstenedione (ng/dl) (panel **a**), estrone (pg/ml) (panel **b**), log of estradiol (pg/ml) (panel **c**), dehydroepiandrosterone (μg/dl) (panel **d**), testosterone (ng/dl) (panel **e**), and the ratio of estrone:androstenedione (panel **f**) by year from the final menstrual period. Red dashed lines indicate concentrations among White women and blue dashed lines indicate concentrations in African-Americans (AAs)

**Fig. 2:**
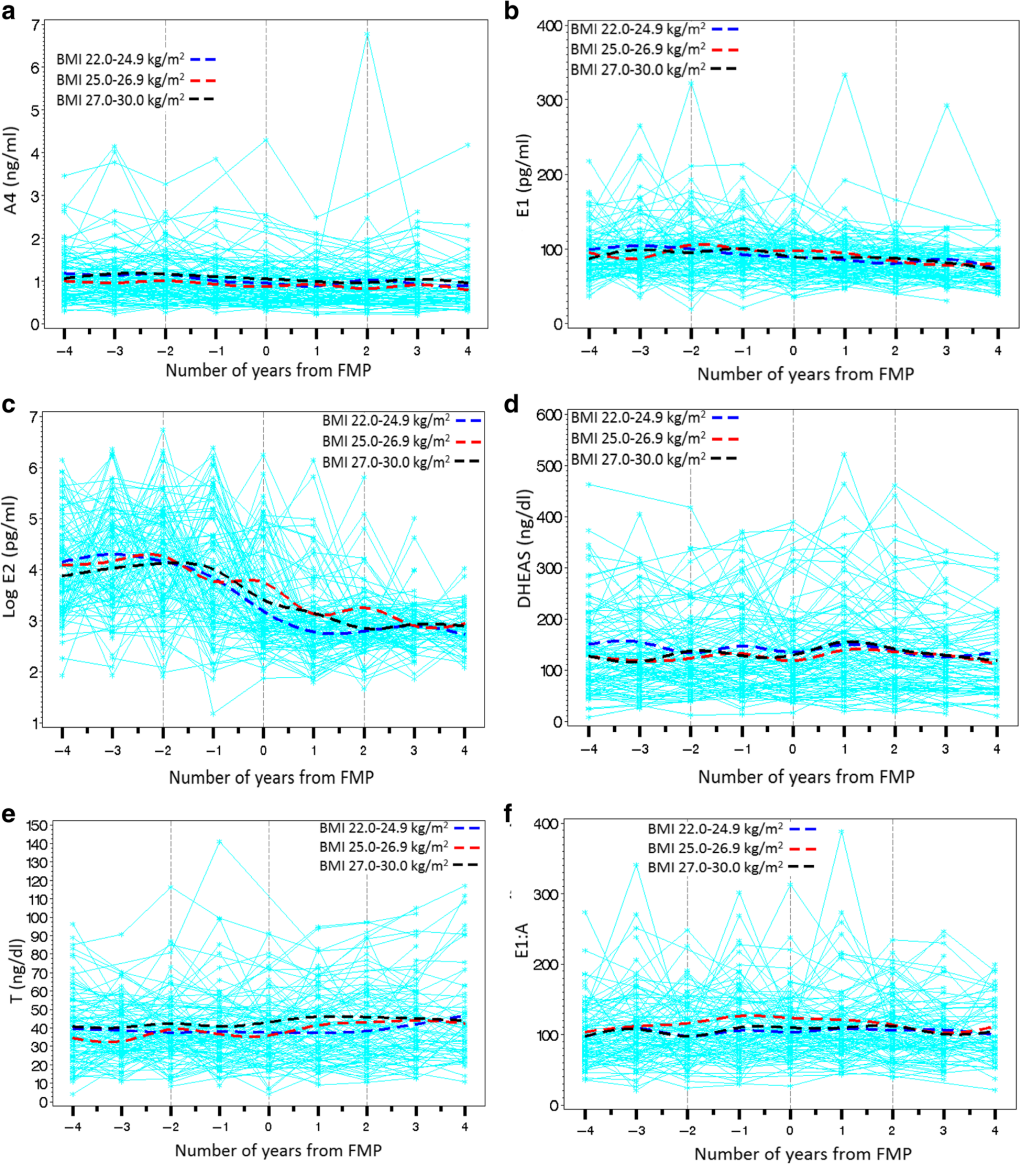
Concentrations of androstenedione (ng/dl) (panel **a**), estrone (pg/ml) (panel **b**), log of estradiol (pg/ml) (panel **c**), dehydroepiandrosterone (μg/dl) (panel **d**), testosterone (ng/dl) (panel **e**), and the ratio of estrone:androstenedione (panel **f**) by year from the final menstrual period. Blue dashed lines indicate concentrations among women with a BMI 22–24.9 kg/m^2^, red dashed lines indicate concentrations among 25–26.9 kg/m^2^, and black dashed lines indicate concentrations in women with a BMI 27–29.9 kg/m^2^

For the purposes of this analysis, BMI was analyzed in tertiles (< 25 kg/m^2^, 25–26.9 kg/m^2^, >27 kg/m^2^) and by White vs. AA race/ethnicity. Distributions of DHEAS, A4, T, E2, and E1 were examined at each year in relation to time before and after the FMP. Population hormone trajectories in relation to FMP and covariates were analyzed using linear mixed models. Piecewise linear mixed models were applied to test the rate of changes at each stage, i.e., pre-menopause (2 years before FMP), transition stage (+/− 2 years around FMP), and post-menopause (2 years after FMP). [2, 14, 15] For presentation in Figs. 1 and 2, data were stratified by race/ethnicity and BMI (normal vs. overweight and by tertile of BMI). In order to determine whether race/ethnicity or BMI was associated with serum hormone concentrations, we created semiparametric stochastic mixed models that accounted for the multiple repeated measures in women and adjusted for the time from the FMP. [16] Hormone distributions were also examined after log-transformation; log transformation did not alter the pattern of the results with the exception of E2, so non-transformed values are presented for other sex hormones. Racial/ethnic differences in SHBG were also examined, but differences were minimal (results not shown). All analyses were performed with SAS Windows 9.2 (SAS Institute, Cary NC).

## Results

Thirty-four AA and 76 White women were included. Participant characteristics are shown in Table 1. Forty-seven (42%) of women had BMIs of 22–24.9 kg/m^2^ vs. 30 (27%) of women who had BMIs 25.0–26.9 kg/m^2^ vs. 33 (30%) who had BMIs 27.0–30.0 kg/m^2^. Women had lower median FSH concentrations at 4 years prior to their FMP (21.4 IU/L) compared to 4 years after their FMP (125.7 IU/L), consistent with the transition from premenopause to postmenopause.

**Table 1:** Characteristics of the study population by race/ethnicity

	White women	African-American women
	(*n* = 76)	(*n* = 34)
Age at baseline (years)	46.36 (2.47)	45.96 (2.05)
Age at final menstrual period (years)	52.03 (2.52)	51.96 (1.97)
Self-reported health (*n*, %)		
Excellent	29 (38.67%)	4 (12.50%)
Very good	36 (48.00%)	9 (28.13%)
Good	8 (10.67%)	15 (46.88%)
Fair/Poor	2 (2.67%)	4 (12.50%)
Body mass index (kg/m^2^)	25.61 (2.18)	25.71 (2.01)
Baseline smoking status (n,%)		
Never	40 (52.63%)	20 (60.61%)
Past	26 (34.21%)	7 (21.21%)
Current	10 (13.16%)	7 (21.21%)

Figure 1 displays the average concentrations of sex hormones across the FMP for White and AA women for each year of the MT. Declines in log E2 concentrations were the most marked out of all of the sex hormone changes in both Whites and AAs, but E2 declines were not accompanied by increases in E1 concentrations. The ratio of E1:A4 remained fairly constant across the MT. Table 2 shows median values for sex hormones at 4 years prior the FMP, the year of the FMP, and 4 years after the FMP for Whites and AAs, and Table 3 shows the association between race/ethnicity and hormone concentration after adjustment for FMP and repeated measures within women. Hormone concentrations were generally higher in Whites than AAs, although only T and E1 met criteria for significance and log E2 concentrations were similar between Whites and AAs.

**Table 2:** Median (interquartile range) serum hormone levels of women 4 years prior to their final menstrual period (FMP), at the time of their FMP, and 4 years after their FMP (*n* = 110)

	4 years prior to FMP	Year of the FMP	4 years after the FMP
DHEAS (μg/dl)	127.5 (100.4)	108.2 (89.1)	98.6 (105.9)
African-American	103.2 (82.4)	104.8 (113.5)	98.2 (66.7)
White	130.2 (109.7)	109.6 (79.2)	102.1 (123.3)
*p*-value	0.116	0.583	0.156
BMI 22.0–24.9 kg/m^2^	133.0 (118.2)	108.2 (92.8)	125.1 (128.8)
BMI 25.0–26.9 kg/m^2^	110.0 (63.1)	110.3 (58.2)	98.2 (70.6)
BMI 27.0–30.0 kg/m^2^	107.2 (69.3)	84.4 (110.8)	95.6 (56.8)
*p*-value	0.316	0.730	0.772
A4 (ng/ml)	1.03 (0.67)	0.84 (0.58)	0.70 (0.59)
African-American	1.03 (0.72)	0.75 (0.33)	0.61 (0.38)
White	1.01 (0.71)	0.91 (0.65)	0.78 (0.62)
* p*-value	0.338	0.066	0.075
BMI 22.0–24.9 kg/m^2^	1.10 (0.70)	0.86 (0.57)	0.77 (0.59)
BMI 25.0–26.9 kg/m^2^	0.99 (0.59)	0.83 (0.52)	0.70 (0.61)
BMI 27.0–30.0 kg/m^2^	0.94 (0.53)	0.81 (0.64)	0.68 (0.58)
*p*-value	0.348	0.800	0.883
T (ng/dl)	35.9 (24.8)	36.3 (21.0)	38.3 (24.4)
African-American	34.6 (18.7)	30.9 (20.0)	33.6 (26.2)
White	36.7 (30.2)	38.7 (20.2)	38.8 (29.6)
*p*-value	0.095	0.020	0.111
BMI 22.0–24.9 kg/m^2^	37.8 (32.8)	36.3 (22.1)	38.8 (24.8)
BMI 25.0–26.9 kg/m^2^	31.2 (18.2)	36.2 (20.5)	40.1 (29.9)
BMI 27.0–30.0 kg/m^2^	36.4 (19.7)	37.2 (28.3)	37.7 (24.2)
*p*-value	0.418	0.566	0.831
E2 (pg/ml)	53.0 (74.8)	21.6 (39.8)	16.6 (8.0)
African-American	53.5 (65.5)	18.7 (10.3)	16.6 (14.9)
White	51.6 (79.8)	24.4 (54.8)	16.6 (7.9)
*p*-value	0.741	0.155	0.692
BMI 22.0–24.9 kg/m^2^	59.5 (99.0)	18.8 (27.4)	14.4 (5.3)
BMI 25.0–26.9 kg/m^2^	41.7 (123.8)	21.0 (127.6)	18.1 (7.0)
BMI 27.0–30.0 kg/m^2^	51.6 (46.6)	24.3 (32.9)	17.5 (9.2)
*p*-value	0.533	0.166	0.090
E1 (pg/ml)	89.4 (39.4)	87.0 (38.6)	69.9 (31.0)
African-American	77.8 (44.5)	85.4 (34.5)	64.8 (30.6)
White	93.4 (38.4)	88.4 (40.4)	72.7 (29.9)
*p*-value	0.069	0.232	0.369
BMI 22.0–24.9 kg/m^2^	97.0 (42.3)	86.9 (45.9)	67.9 (23.9)
BMI 25.0–26.9 kg/m^2^	94.4 (41.8)	100.0 (42.0)	82.8 (42.9)
BMI 27.0–30.0 kg/m^2^	80.5 (26.7)	80.3 (34.2)	65.5 (34.5)
*p*-value	0.111	0.317	0.633

**Table 3:** Associations between race/ethnicity and hormone levels from semiparametric stochastic mixed models, beta-coefficient (standard error) and *p*-value

	Beta-coefficient (standard error)	*p*-value
DHEAS (μg/dl)	28.80 (15.34)	0.061
A4 (ng/ml)	0.2556 (0.1315)	0.052
T (ng/dl)	9.180 (1.652)	<0.00001
ln E2	0.0764 (0.0695)	0.272
E1 (pg/ml)	11.365 (0.7306)	<0.00001
E1:A4	−6.527 (7.040)	0.354

Reference group is African-American women; a beta-coefficient greater than 0 indicates higher sex hormone levels in white women

Figure 2 shows the average concentration trajectories of sex hormones across the MT for women by BMI tertile. Table 2 shows median values for sex hormones at 4 years prior the FMP, the year of the FMP, and 4 years after the FMP by BMI tertile. BMI tertile was not associated with differences in DHEAS, A4, or E1 at different times in relation to the FMP. Table 4 shows the association between BMI as a continuous variable and hormone concentration after adjustment for FMP and repeated measures within women. Although BMI as a continuous variable was associated with slightly higher T concentrations, this association was of borderline statistical significance (*p* = 0.051). Otherwise, higher BMI was not associated with higher hormone concentrations.

**Table 4:** Associations between body mass index (BMI) and hormone levels from semiparametric stochastic mixed models, beta-coefficient (standard error) is the unit hormone increase per kg/m^2^

	Beta-coefficient (standard error)	*p*-value
DHEAS (μg/dl)	0.0000 (0.0000)	1.00
A4 (ng/ml)	−0.0026 (0.0289)	0.929
T (ng/dl)	0.7359 (0.3758)	0.051
ln E2	0.0230 (0.0151)	0.128
E1 (pg/ml)	−0.3466 (0.912)	0.704
E1:A4	0.0274 (1.5409)	0.354

## Discussion

In a longitudinal analysis spanning 8 years across the MT, DHEAS concentrations were stable across the MT [6, 7]. We also note that A4, T, and E1 concentrations remain relatively stable as long as 4 years after the FMP. Moreover, the ratios of E1 and A4 remained fairly constant across the MT. Although A4 and E1 declined slightly, these changes did not mirror the dramatic declines in E2 production. We also found that AAs had slightly lower sex hormone concentrations than Whites. The racial/ethnic differences were likely not due to BMI, as the nature and rate of decline in DHEAS, A4, and E1 were similar by BMI. Our results are consistent with previous reports that suggest that a rise in DHEAS concentrations prior to menopause is concurrent with the declines in peripheral levels of other sex steroids, as well as reports that note declines in DHEAS after the FMP [6–10]. Our report is novel in its inclusion of AAs, the breadth of sex hormones examined, the longitudinal analysis of androgens timed to the FMP, and the length of time spanning the MT.

With the marked decline in ovarian estrogen production in the postmenopause, the adrenal gland becomes a particularly important source of sex steroids, chiefly androgens. DHEA produced by the ovary and the adrenal gland is aromatized peripherally via A4 to bioactive estrogens such as E1. The adrenal gland is also an important source of T which is aromatized to E2. Adrenal-ovarian sex hormone production, conversion to other hormones, and clearance have been postulated to exist in equilibrium [6]. However, longitudinal studies testing this hypothesis are few. Previous reports in SWAN have noted that the majority of women had slight increases in DHEAS prior the FMP followed by declines after the FMP [6, 7], raising the possibility that increased production of DHEAS and/or conversion to other androgens could be a contributor to women’s increased androgenicity postmenopause.

Our report extends this prior work: we confirm a modest perimenopausal increase in DHEAS levels, stable T concentrations, and minimal declines in A4 up to 4 years after the FMP. Additionally, since DHEAS and A4 changes do not mirror each other over the transition, it is unlikely that the fluctuations in DHEAS are due to increased peripheral conversion into A4. Similarly, as E1:A4 ratios were stable over time, it is also unlikely that aromatization of A4 to E1 changes significantly over the MT, assuming that A4 production remains the same, although it is possible that both A4 production and aromatization increased concomitantly. Other reports have not reported changes in DHEAS, A4, and T across the menopause. In a cohort of 59 Norwegian women, Overlie and colleagues noted that A4 levels corresponded with both E1 and E2 levels, consistent with the shift from ovarian to adrenal sex hormone production in the postmenopause. However, A4 levels declined in the premenopause, and no significant changes were observed in DHEAS [17]. In a cohort of Swedish women, Rannevik and colleagues also observed that A4 correlated with postmenopausal E1 and E2 concentrations, but concentrations of DHEAS and A4 declined minimally [18].

Our report and previous SWAN studies may have found modest perimenopausal elevations in DHEAS due to larger sample size and our ability to follow women over a longer period of time prior to and after the FMP. Thus, changes in the estrogen/androgen ratio are driven by declines in circulating estrogens, and this relative increase in androgenicity may drive some of the phenotypic changes characteristic of later menopause, such as increased hirsutism [19]. Although speculative, it is possible that such effects are exerted at the tissue level, due to the differential binding of A4 and E2 [20]. Changes in the estrogen/androgen ratio may also be driven by the adrenal response to changing LH concentrations: studies in humans have noted the presence of luteinizing hormone (LH) receptors in the adrenal cortex [21], and mouse models note increases in LH receptors in response to increasing LH levels [22], thus explaining how the adrenal gland might increase sex hormone production even as ovarian response to LH declines.

Previous publications have also reported racial/ethnic differences in androgens, although the direction of reported associations is inconsistent. In a cross-sectional study, Spencer and colleagues noted that even after adjustment for age, BMI, and insulin resistance, AA women had lower DHEAS, A4, and T concentrations than White women [23]. In contrast, Kim and colleagues noted minimal differences in a glucose-intolerant population [24]. Thus, the source of racial/ethnic differences in DHEAS, A4, E1, and T remain speculative. Previous analyses using SWAN data have noted that AAs had the lowest overall DHEAS levels and lowest rates of decline with chronologic age [6, 7]. In those reports, concentrations of A4 were not examined, and thus it was unclear whether racial/ethnic differences in DHEAS concentrations were due to increased adrenal androgen production vs. decreased metabolism of DHEAS into A4. Our report suggests that racial/ethnic differences in A4 metabolism are unlikely to contribute to racial/ethnic differences in DHEAS concentrations.

Previous reports have also suggested that weight may be a significant modifier of adrenal sex hormone production, possibly by affecting E2 concentrations, which in turn might lead to compensatory increases in E1. However, we did not find significant effect modification by tertile of BMI for the sex hormones examined in this report. Our report agrees with that of population-based studies examining cross-sectional associations between DHEAS, A4, and BMI in postmenopausal women [25, 26]. Although E2 and T correlate with waist circumference and BMI in women, the association between BMI and other sex hormones has been relatively weak. One explanation for our conflicting results is that we examined non-obese women within a narrow range of BMI. It is also possible that BMI and waist circumference do not reflect adipose tissue deposition, as examination of associations between visceral adiposity and sex steroids using radiographic imaging has found stronger associations [27].

Strengths of the current report include the longitudinal design, inclusion of AAs, examination of a comprehensive list of adrenal sex hormones, high assay sensitivity for low androgen concentrations, and observation for 9 years during the MT for 990 observations. Limitations include a limited sample size, and thus small fluctuations in hormone concentrations may not have been detected. Our ability to adjust for confounders, particularly racial-ethnic differences in adipose tissue deposition, was also limited. It is possible that self-rated health and smoking status contributed to racial/ethnic differences along with unmeausured confounders, but we had limited power to adjust for these possibilities. We did not use LC/MS for measurement of E2 concentrations, which are low after the FMP; however, our objective was to show relative change over the MT, rather than to establish a definitive absolute value for E2. Finally, we did not conduct adrenal and ovarian vein sampling, and thus cannot definitively distinguish between ovarian and adrenal production of androgens and estrogens.

## Conclusions

Our report supports the importance of adrenal androgens as the primary source of estrogens in the postmenopause and the increased androgenicity of the postmenopausal hormonal milieu. It is also possible that ovarian production of E1 remains even as E2 declines. Modest increases in DHEAS concentrations are not accompanied by measurably increased levels of A4, T, or E1. Concentrations of these hormones appear to be lower in AAs than White women in the perimenopause, and these racial/ethnic differences are unlikely due to BMI. Examination of the mechanisms for lower DHEAS, A4, and T concentrations in AA women is needed, particularly prior the FMP when declines in other sex hormones occur. Examination of whether these differences contribute to vasomotor symptoms or altered risk of chronic disease risk by race/ethnicity, particularly in other groups besides Whites and AAs, is needed.

## Abbreviations

A4: Androstenedione
AAs: African-Americans
BMI: Body mass index
DHEAS: Dehydroepiandrosterone sulfate
E1: Estrone
E2: Estradiol
FMP: Follicle stimulating hormone
FSH: Follicle stimulating hormone
LC/MS: Liquid chromatography mass spectrometry
LH: Luteinizing hormone
MT: Menopausal transition
T: Testosterone
